# Neighborhood Poverty Exposure in Adulthood and Cognitive Outcomes in Later Life

**DOI:** 10.1093/geroni/igaf122.1297

**Published:** 2025-12-31

**Authors:** Lucie Kalousova

**Affiliations:** Vanderbilt University, Nashville, Tennessee, United States

## Abstract

Dementia risk is shaped not only by biological and individual factors but also by broader structural conditions, including neighborhood disadvantage. I examine the long-term impact of neighborhood poverty on cognitive health, focusing on when in the life course exposure matters most. Using longitudinal data from the Panel Study of Income Dynamics (PSID: N = 1,957), I reconstruct respondents’ neighborhood poverty trajectories from early adulthood to late life. I apply sequence analysis to classify individuals into distinct patterns of exposure and use marginal structural models with inverse probability of treatment weights to estimate the effects of neighborhood poverty on dementia risk while accounting for time-varying confounding. My findings show that cumulative exposure to neighborhood poverty is associated with higher odds of cognitive impairment in later life (OR: 1.39; [1.28, 1.51]. Early adulthood exposure appears more consequential (OR: 1.24; [1.06, 1.45]) than later life exposure (OR:1.05; [0.98, 1.13]. The results suggest that interventions aimed at reducing dementia risk must begin earlier in the life course and contribute to the growing literature on the social determinants of cognitive health by clarifying the role of neighborhood disadvantage across different life stages. They also highlight the need for policies that address structural inequalities in housing and neighborhood conditions long before cognitive decline becomes apparent.

